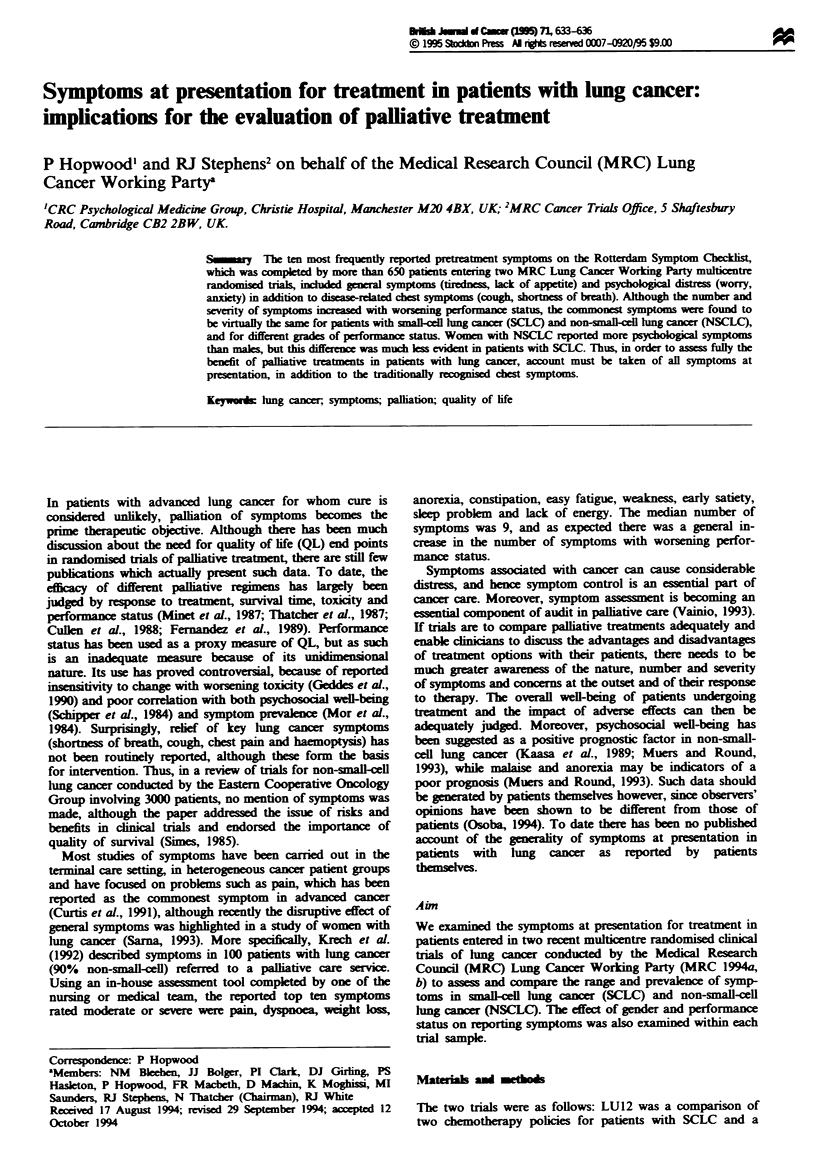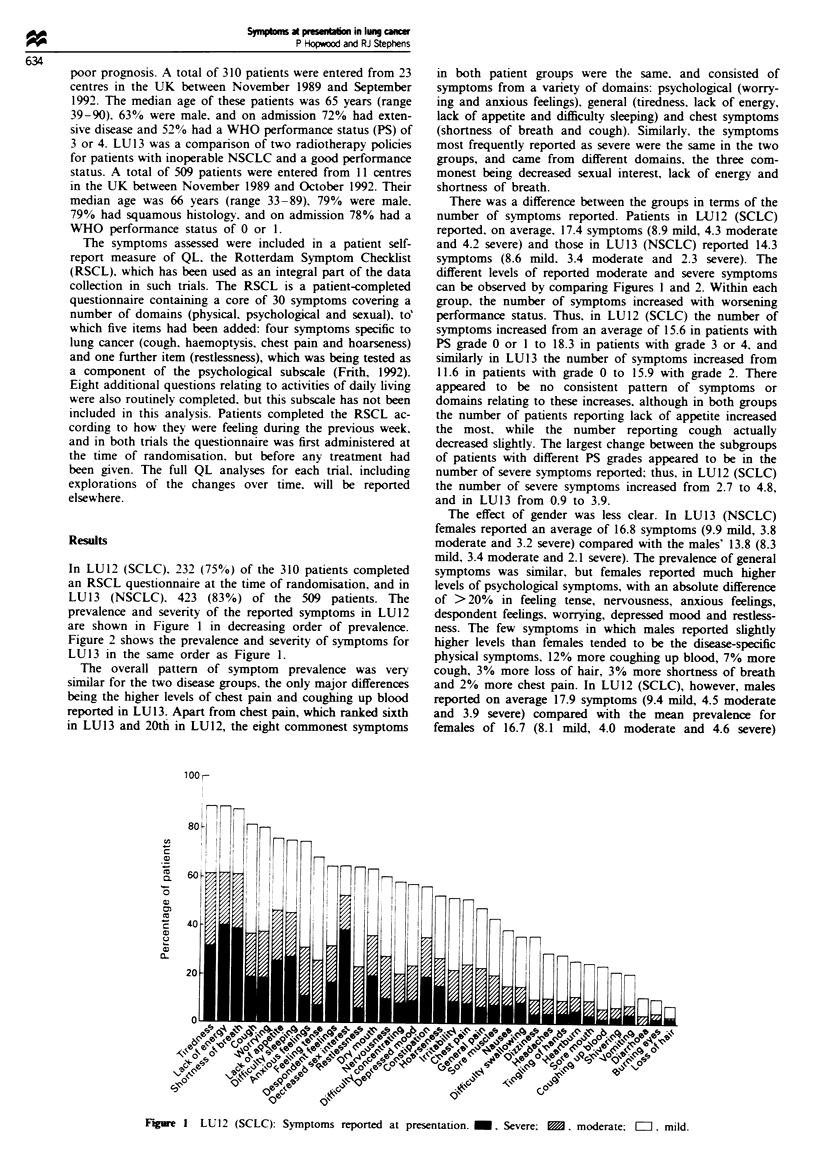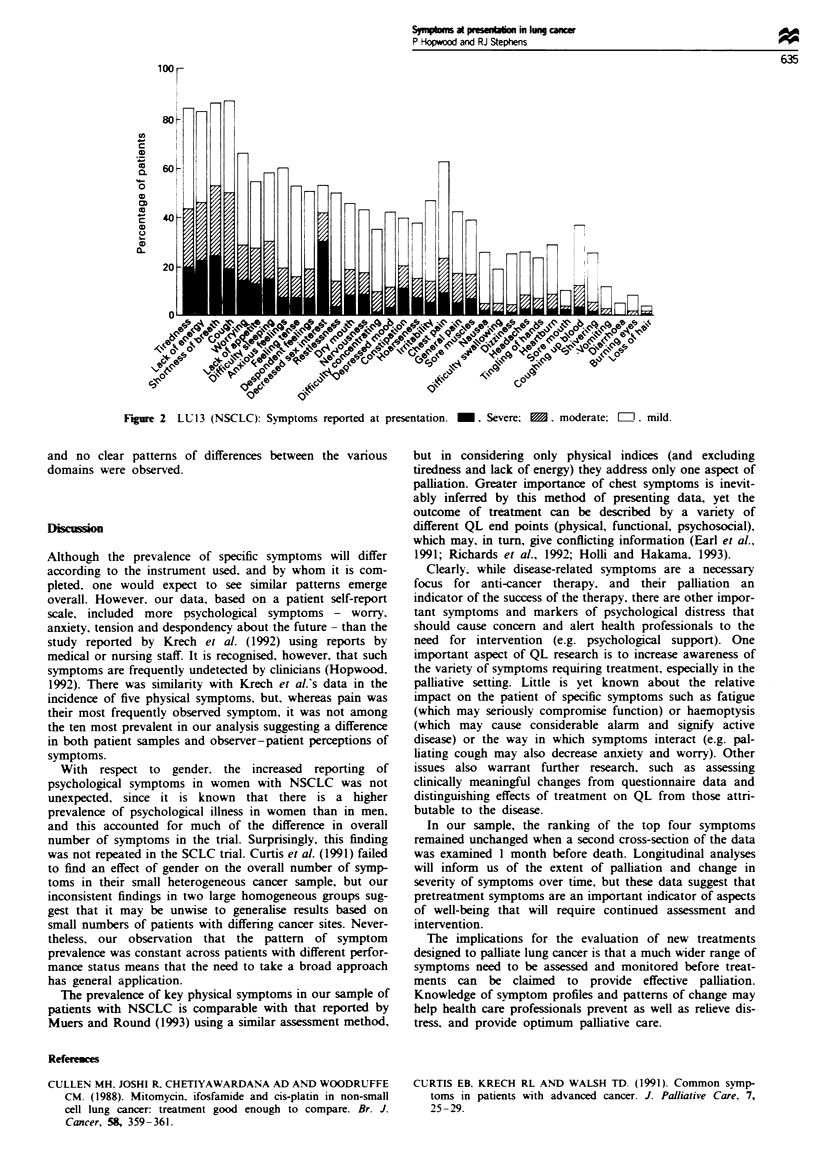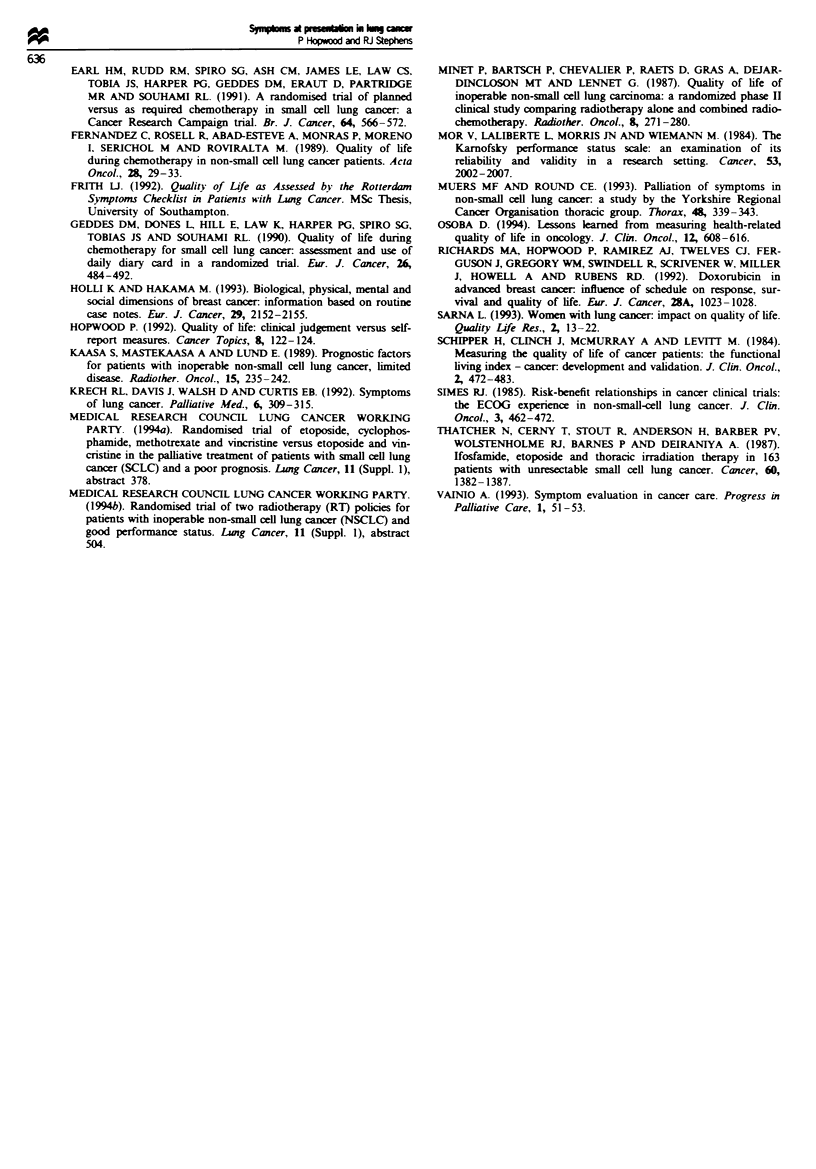# Symptoms at presentation for treatment in patients with lung cancer: implications for the evaluation of palliative treatment. The Medical Research Council (MRC) Lung Cancer Working Party.

**DOI:** 10.1038/bjc.1995.124

**Published:** 1995-03

**Authors:** P. Hopwood, R. J. Stephens

**Affiliations:** CRC Psychological Medicine Group, Christie Hospital, Manchester, UK.

## Abstract

The ten most frequently reported pretreatment symptoms on the Rotterdam Symptom Checklist, which was completed by more than 650 patients entering two MRC Lung Cancer Working Party multicentre randomised trials, included general symptoms (tiredness, lack of appetite) and psychological distress (worry, anxiety) in addition to disease-related chest symptoms (cough, shortness of breath). Although the number and severity of symptoms increased with worsening performance status, the commonest symptoms were found to be virtually the same for patients with small-cell lung cancer (SCLC) and non-small-cell lung cancer (NSCLC), and for different grades of performance status. Women with NSCLC reported more psychological symptoms than males, but this difference was much less evident in patients with SCLC. Thus, in order to assess fully the benefit of palliative treatments in patients with lung cancer, account must be taken of all symptoms at presentation, in addition to the traditionally recognised chest symptoms.


© 1995 Stockton Press All rights reserved 0007-0920/95 $9.00

### Symptoms at presentation for treatment in patients with lung cancer: implications for the evaluation of palliative treatment

P Hopwood[1] and RJ Stephens[2] on behalf of the Medical Research Council (MRC) Lung Cancer Working Party[a]

[1]*CRC Psychological Medicine Group, Christie Hospital, Manchester M20 4BX, UK;* [2]*MRC Cancer Trials Office, 5 Shaftesbury Road, Cambridge CB2 2BW, UK.*

**Summary** The ten most frequently reported pretreatment symptoms on the Rotterdam Symptom Checklist, which was completed by more than 650 patients entering two MRC Lung Cancer Working Party multicentre randomised trials, included general symptoms (tiredness, lack of appetite) and psychological distress (worry, anxiety) in addition to disease-related chest symptoms (cough, shortness of breath). Although the number and severity of symptoms increased with worsening performance status, the commonest symptoms were found to be virtually the same for patients with small-cell lung cancer (SCLC) and non-small-cell lung cancer (NSCLC), and for different grades of performance status. Women with NSCLC reported more psychological symptoms than males, but this difference was much less evident in patients with SCLC. Thus, in order to assess fully the benefit of palliative treatments in patients with lung cancer, account must be taken of all symptoms at presentation, in addition to the traditionally recognised chest symptoms.

**Keywords:** lung cancer; symptoms; palliation; quality of life

In patients with advanced lung cancer for whom cure is considered unlikely, palliation of symptoms becomes the prime therapeutic objective. Although there has been much discussion about the need for quality of life (QL) end points in randomised trials of palliative treatment, there are still few publications which actually present such data. To date, the efficacy of different palliative regimens has largely been judged by response to treatment, survival time, toxicity and performance status (Minet *et al.*, 1987; Thatcher *et al.*, 1987; Cullen *et al.*, 1988; Fernandez *et al.*, 1989). Performance status has been used as a proxy measure of QL, but as such is an inadequate measure because of its unidimensional nature. Its use has proved controversial, because of reported insensitivity to change with worsening toxicity (Geddes *et al.*, 1990) and poor correlation with both psychosocial well-being (Schipper *et al.*, 1984) and symptom prevalence (Mor *et al.*, 1984). Surprisingly, relief of key lung cancer symptoms (shortness of breath, cough, chest pain and haemoptysis) has not been routinely reported, although these form the basis for intervention. Thus, in a review of trials for non-small-cell lung cancer conducted by the Eastern Cooperative Oncology Group involving 3000 patients, no mention of symptoms was made, although the paper addressed the issue of risks and benefits in clinical trials and endorsed the importance of quality of survival (Simes, 1985).

Most studies of symptoms have been carried out in the terminal care setting, in heterogeneous cancer patient groups and have focused on problems such as pain, which has been reported as the commonest symptom in advanced cancer (Curtis *et al.*, 1991), although recently the disruptive effect of general symptoms was highlighted in a study of women with lung cancer (Sarna, 1993). More specifically, Krech *et al.* (1992) described symptoms in 100 patients with lung cancer (90% non-small-cell) referred to a palliative care service. Using an in-house assessment tool completed by one of the nursing or medical team, the reported top ten symptoms rated moderate or severe were pain, dyspnoea, weight loss, anorexia, constipation, easy fatigue, weakness, early satiety, sleep problem and lack of energy. The median number of symptoms was 9, and as expected there was a general increase in the number of symptoms with worsening performance status.

Symptoms associated with cancer can cause considerable distress, and hence symptom control is an essential part of cancer care. Moreover, symptom assessment is becoming an essential component of audit in palliative care (Vainio, 1993). If trials are to compare palliative treatments adequately and enable clinicians to discuss the advantages and disadvantages of treatment options with their patients, there needs to be much greater awareness of the nature, number and severity of symptoms and concerns at the outset and of their response to therapy. The overall well-being of patients undergoing treatment and the impact of adverse effects can then be adequately judged. Moreover, psychosocial well-being has been suggested as a positive prognostic factor in non-small-cell lung cancer (Kaasa *et al.*, 1989; Muers and Round, 1993), while malaise and anorexia may be indicators of a poor prognosis (Muers and Round, 1993). Such data should be generated by patients themselves however, since observers' opinions have been shown to be different from those of patients (Osoba, 1994). To date there has been no published account of the generality of symptoms at presentation in patients with lung cancer as reported by patients themselves.

### *Aim*

We examined the symptoms at presentation for treatment in patients entered in two recent multicentre randomised clinical trials of lung cancer conducted by the Medical Research Council (MRC) Lung Cancer Working Party (MRC 1994*a*, *b*) to assess and compare the range and prevalence of symptoms in small-cell lung cancer (SCLC) and non-small-cell lung cancer (NSCLC). The effect of gender and performance status on reporting symptoms was also examined within each trial sample.

### Materials and methods

The two trials were as follows: LU12 was a comparison of two chemotherapy policies for patients with SCLC and a

Correspondence: P Hopwood

[a]Members: NM Bleehen, JJ Bolger, PI Clark, DJ Girling, PS Hasleton, P Hopwood, FR Macbeth, D Machin, K Moghissi, MI Saunders, RJ Stephens, N Thatcher (Chairman), RJ White

Received 17 August 1994; revised 29 September 1994; accepted 12 October 1994

poor prognosis. A total of 310 patients were entered from 23 centres in the UK between November 1989 and September 1992. The median age of these patients was 65 years (range 39–90), 63% were male, and on admission 72% had extensive disease and 52% had a WHO performance status (PS) of 3 or 4. LU13 was a comparison of two radiotherapy policies for patients with inoperable NSCLC and a good performance status. A total of 509 patients were entered from 11 centres in the UK between November 1989 and October 1992. Their median age was 66 years (range 33–89), 79% were male, 79% had squamous histology, and on admission 78% had a WHO performance status of 0 or 1.

The symptoms assessed were included in a patient self-report measure of QL, the Rotterdam Symptom Checklist (RSCL), which has been used as an integral part of the data collection in such trials. The RSCL is a patient-completed questionnaire containing a core of 30 symptoms covering a number of domains (physical, psychological and sexual), to which five items had been added: four symptoms specific to lung cancer (cough, haemoptysis, chest pain and hoarseness) and one further item (restlessness), which was being tested as a component of the psychological subscale (Frith, 1992). Eight additional questions relating to activities of daily living were also routinely completed, but this subscale has not been included in this analysis. Patients completed the RSCL according to how they were feeling during the previous week, and in both trials the questionnaire was first administered at the time of randomisation, but before any treatment had been given. The full QL analyses for each trial, including explorations of the changes over time, will be reported elsewhere.

### Results

In LU12 (SCLC), 232 (75%) of the 310 patients completed an RSCL questionnaire at the time of randomisation, and in LU13 (NSCLC), 423 (83%) of the 509 patients. The prevalence and severity of the reported symptoms in LU12 are shown in Figure 1 in decreasing order of prevalence. Figure 2 shows the prevalence and severity of symptoms for LU13 in the same order as Figure 1.

The overall pattern of symptom prevalence was very similar for the two disease groups, the only major differences being the higher levels of chest pain and coughing up blood reported in LU13. Apart from chest pain, which ranked sixth in LU13 and 20th in LU12, the eight commonest symptoms in both patient groups were the same, and consisted of symptoms from a variety of domains: psychological (worrying and anxious feelings), general (tiredness, lack of energy, lack of appetite and difficulty sleeping) and chest symptoms (shortness of breath and cough). Similarly, the symptoms most frequently reported as severe were the same in the two groups, and came from different domains, the three commonest being decreased sexual interest, lack of energy and shortness of breath.

There was a difference between the groups in terms of the number of symptoms reported. Patients in LU12 (SCLC) reported, on average, 17.4 symptoms (8.9 mild, 4.3 moderate and 4.2 severe) and those in LU13 (NSCLC) reported 14.3 symptoms (8.6 mild, 3.4 moderate and 2.3 severe). The different levels of reported moderate and severe symptoms can be observed by comparing Figures 1 and 2. Within each group, the number of symptoms increased with worsening performance status. Thus, in LU12 (SCLC) the number of symptoms increased from an average of 15.6 in patients with PS grade 0 or 1 to 18.3 in patients with grade 3 or 4, and similarly in LU13 the number of symptoms increased from 11.6 in patients with grade 0 to 15.9 with grade 2. There appeared to be no consistent pattern of symptoms or domains relating to these increases, although in both groups the number of patients reporting lack of appetite increased the most, while the number reporting cough actually decreased slightly. The largest change between the subgroups of patients with different PS grades appeared to be in the number of severe symptoms reported; thus, in LU12 (SCLC) the number of severe symptoms increased from 2.7 to 4.8, and in LU13 from 0.9 to 3.9.

The effect of gender was less clear. In LU13 (NSCLC) females reported an average of 16.8 symptoms (9.9 mild, 3.8 moderate and 3.2 severe) compared with the males' 13.8 (8.3 mild, 3.4 moderate and 2.1 severe). The prevalence of general symptoms was similar, but females reported much higher levels of psychological symptoms, with an absolute difference of >20% in feeling tense, nervousness, anxious feelings, despondent feelings, worrying, depressed mood and restlessness. The few symptoms in which males reported slightly higher levels than females tended to be the disease-specific physical symptoms, 12% more coughing up blood, 7% more cough, 3% more loss of hair, 3% more shortness of breath and 2% more chest pain. In LU12 (SCLC), however, males reported on average 17.9 symptoms (9.4 mild, 4.5 moderate and 3.9 severe) compared with the mean prevalence for females of 16.7 (8.1 mild, 4.0 moderate and 4.6 severe)

**Figure 1** LU12 (SCLC): Symptoms reported at presentation. ■, Severe; ▨, moderate; □, mild.

**Figure 2** LU13 (NSCLC): Symptoms reported at presentation. ■, Severe; ▨, moderate; □, mild.

and no clear patterns of differences between the various domains were observed.

### Discussion

Although the prevalence of specific symptoms will differ according to the instrument used, and by whom it is completed, one would expect to see similar patterns emerge overall. However, our data, based on a patient self-report scale, included more psychological symptoms – worry, anxiety, tension and despondency about the future – than the study reported by Krech *et al.* (1992) using reports by medical or nursing staff. It is recognised, however, that such symptoms are frequently undetected by clinicians (Hopwood, 1992). There was similarity with Krech *et al.*'s data in the incidence of five physical symptoms, but, whereas pain was their most frequently observed symptom, it was not among the ten most prevalent in our analysis suggesting a difference in both patient samples and observer–patient perceptions of symptoms.

With respect to gender, the increased reporting of psychological symptoms in women with NSCLC was not unexpected, since it is known that there is a higher prevalence of psychological illness in women than in men, and this accounted for much of the difference in overall number of symptoms in the trial. Surprisingly, this finding was not repeated in the SCLC trial. Curtis *et al.* (1991) failed to find an effect of gender on the overall number of symptoms in their small heterogeneous cancer sample, but our inconsistent findings in two large homogeneous groups suggest that it may be unwise to generalise results based on small numbers of patients with differing cancer sites. Nevertheless, our observation that the pattern of symptom prevalence was constant across patients with different performance status means that the need to take a broad approach has general application.

The prevalence of key physical symptoms in our sample of patients with NSCLC is comparable with that reported by Muers and Round (1993) using a similar assessment method, but in considering only physical indices (and excluding tiredness and lack of energy) they address only one aspect of palliation. Greater importance of chest symptoms is inevitably inferred by this method of presenting data, yet the outcome of treatment can be described by a variety of different QL end points (physical, functional, psychosocial), which may, in turn, give conflicting information (Earl *et al.*, 1991; Richards *et al.*, 1992; Holli and Hakama, 1993).

Clearly, while disease-related symptoms are a necessary focus for anti-cancer therapy, and their palliation an indicator of the success of the therapy, there are other important symptoms and markers of psychological distress that should cause concern and alert health professionals to the need for intervention (e.g. psychological support). One important aspect of QL research is to increase awareness of the variety of symptoms requiring treatment, especially in the palliative setting. Little is yet known about the relative impact on the patient of specific symptoms such as fatigue (which may seriously compromise function) or haemoptysis (which may cause considerable alarm and signify active disease) or the way in which symptoms interact (e.g. palliating cough may also decrease anxiety and worry). Other issues also warrant further research, such as assessing clinically meaningful changes from questionnaire data and distinguishing effects of treatment on QL from those attributable to the disease.

In our sample, the ranking of the top four symptoms remained unchanged when a second cross-section of the data was examined 1 month before death. Longitudinal analyses will inform us of the extent of palliation and change in severity of symptoms over time, but these data suggest that pretreatment symptoms are an important indicator of aspects of well-being that will require continued assessment and intervention.

The implications for the evaluation of new treatments designed to palliate lung cancer is that a much wider range of symptoms need to be assessed and monitored before treatments can be claimed to provide effective palliation. Knowledge of symptom profiles and patterns of change may help health care professionals prevent as well as relieve distress, and provide optimum palliative care.

### References

CULLEN MH, JOSHI R, CHETIYAWARDANA AD AND WOODRUFFE CM. (1988). Mitomycin, ifosfamide and cis-platin in non-small cell lung cancer: treatment good enough to compare. *Br. J. Cancer*, **58**, 359–361.

CURTIS EB, KRECH RL AND WALSH TD. (1991). Common symptoms in patients with advanced cancer. *J. Palliative Care*, **7**, 25–29.

EARL HM, RUDD RM, SPIRO SG, ASH CM, JAMES LE, LAW CS, TOBIA JS, HARPER PG, GEDDES DM, ERAUT D, PARTRIDGE MR AND SOUHAMI RL. (1991). A randomised trial of planned versus as required chemotherapy in small cell lung cancer: a Cancer Research Campaign trial. *Br. J. Cancer*, **64**, 566-572.

FERNANDEZ C, ROSELL R, ABAD-ESTEVE A, MONRAS P, MORENO I, SERICHOL M AND ROVIRALTA M. (1989). Quality of life during chemotherapy in non-small cell lung cancer patients. *Acta Oncol.*, **28**, 29-33.

FRITH LJ. (1992). *Quality of Life as Assessed by the Rotterdam Symptoms Checklist in Patients with Lung Cancer.* MSc Thesis, University of Southampton.

GEDDES DM, DONES L, HILL E, LAW K, HARPER PG, SPIRO SG, TOBIAS JS AND SOUHAMI RL. (1990). Quality of life during chemotherapy for small cell lung cancer: assessment and use of daily diary card in a randomized trial. *Eur. J. Cancer*, **26**, 484-492.

HOLLI K AND HAKAMA M. (1993). Biological, physical, mental and social dimensions of breast cancer: information based on routine case notes. *Eur. J. Cancer*, **29**, 2152-2155.

HOPWOOD P. (1992). Quality of life: clinical judgement versus self-report measures. *Cancer Topics*, **8**, 122-124.

KAASA S, MASTEKAASA A AND LUND E. (1989). Prognostic factors for patients with inoperable non-small cell lung cancer, limited disease. *Radiother. Oncol.*, **15**, 235-242.

KRECH RL, DAVIS J, WALSH D AND CURTIS EB. (1992). Symptoms of lung cancer. *Palliative Med.*, **6**, 309-315.

MEDICAL RESEARCH COUNCIL LUNG CANCER WORKING PARTY. (1994*a*). Randomised trial of etoposide, cyclophosphamide, methotrexate and vincristine versus etoposide and vincristine in the palliative treatment of patients with small cell lung cancer (SCLC) and a poor prognosis. *Lung Cancer*, **11** (Suppl. 1), abstract 378.

MEDICAL RESEARCH COUNCIL LUNG CANCER WORKING PARTY. (1994*b*). Randomised trial of two radiotherapy (RT) policies for patients with inoperable non-small cell lung cancer (NSCLC) and good performance status. *Lung Cancer*, **11** (Suppl. 1), abstract 504.

MINET P, BARTSCH P, CHEVALIER P, RAETS D, GRAS A, DEJARDINCLOSON MT AND LENNET G. (1987). Quality of life of inoperable non-small cell lung carcinoma: a randomized phase II clinical study comparing radiotherapy alone and combined radiochemotherapy. *Radiother. Oncol.*, **8**, 271-280.

MOR V, LALIBERTE L, MORRIS JN AND WIEMANN M. (1984). The Karnofsky performance status scale: an examination of its reliability and validity in a research setting. *Cancer*, **53**, 2002-2007.

MUERS MF AND ROUND CE. (1993). Palliation of symptoms in non-small cell lung cancer: a study by the Yorkshire Regional Cancer Organisation thoracic group. *Thorax*, **48**, 339-343.

OSOBA D. (1994). Lessons learned from measuring health-related quality of life in oncology. *J. Clin. Oncol.*, **12**, 608-616.

RICHARDS MA, HOPWOOD P, RAMIREZ AJ, TWELVES CJ, FERGUSON J, GREGORY WM, SWINDELL R, SCRIVENER W, MILLER J, HOWELL A AND RUBENS RD. (1992). Doxorubicin in advanced breast cancer: influence of schedule on response, survival and quality of life. *Eur. J. Cancer*, **28A**, 1023-1028.

SARNA L. (1993). Women with lung cancer: impact on quality of life. *Quality Life Res.*, **2**, 13-22.

SCHIPPER H, CLINCH J, MCMURRAY A AND LEVITT M. (1984). Measuring the quality of life of cancer patients: the functional living index - cancer: development and validation. *J. Clin. Oncol.*, **2**, 472-483.

SIMES RJ. (1985). Risk-benefit relationships in cancer clinical trials: the ECOG experience in non-small-cell lung cancer. *J. Clin. Oncol.*, **3**, 462-472.

THATCHER N, CERNY T, STOUT R, ANDERSON H, BARBER PV, WOLSTENHOLME RJ, BARNES P AND DEIRANIYA A. (1987). Ifosfamide, etoposide and thoracic irradiation therapy in 163 patients with unresectable small cell lung cancer. *Cancer*, **60**, 1382-1387.

VAINIO A. (1993). Symptom evaluation in cancer care. *Progress in Palliative Care*, **1**, 51-53.